# “To enroll or not to enroll”: a qualitative study on preferences for dental insurance in Iran

**DOI:** 10.1186/s12913-022-08285-8

**Published:** 2022-07-11

**Authors:** Jamileh Vahidi, Amirhossein Takian, Mostafa Amini-Rarani, Maryam Moeeni

**Affiliations:** 1 School of Management & Medical Information Sciences, Isfahan University of Medical Sciences, Isfahan, Iran; 2 Baharloo Hospital, Tehran University of Medical Sciences, Tehran, Iran; 3 Department of Health Management, Policy and Economics, School of Public Health, Tehran University of Medical Sciences, Tehran, Iran; 4 Department of Global Health & Public Policy, School of Public Health, Tehran University of Medical Sciences, Tehran, Iran; 5 Health Equity Research Center (HERC), Tehran University of Medical Sciences, Tehran, Iran; 6 Health Management and Economics Research Center, Isfahan University of Medical Sciences, Isfahan, Iran; 7 Social Determinants of Health Research Center, Isfahan University of Medical Sciences, Isfahan, Iran

**Keywords:** Dental insurance, Health insurance, Preference, Households, Iran

## Abstract

**Background:**

Oral public health services are included in primary healthcare. Although oral diseases are preventable, improving oral health has become a concern in many countries. Evidence shows that functioning insurance coverage can significantly increase the use of dental health services, improve quality of services, and reduce financial barriers to utilization. Little evidence exists on households’ preferences for dental insurance in Iran. This study seeks to identify the households’ preferences for dental insurance in Tehran-Iran.

**Method:**

This is a qualitative study. We interviewed 84 participants who visited selected public and private dental clinics in Tehran-Iran, from October 2018 until January 2019. All interviews were recorded and transcribed verbatim. We used a mixed inductive/deductive approach for thematic analysis of the interviews.

**Results:**

We identified two main themes and 12 sub-themes: pecuniary attributes (insurance premium, coinsurance, insurance coverage granted, discounting option, reimbursement of expenses), and non-pecuniary attributes (notification status, ethical issues, benefits package, contract providers with health insurance, quality of service centers, administrative process, and dental insurance scheme).

**Conclusion:**

Our participants considered both pecuniary and non-pecuniary attributes for choosing a dental insurance package. Our findings could help, we envisage, policymakers understand Iranian households’ preferences for a dental insurance scheme that they afford to buy.

**Supplementary Information:**

The online version contains supplementary material available at 10.1186/s12913-022-08285-8.

## Background

Although oral diseases are largely preventable, improving oral health has become a concern in many countries. Sixty to 90% of school-aged children and almost all adults are suffering from dental problems worldwide. The situation is even worse among the Eastern Mediterranean Region (EMR) countries, i.e. Iran [1, 2]. Over 50 million hours of school time are lost every year because of oral diseases [1, 3]. Childhood malnutrition, social communication disorder, and probability of systemic diseases are only a part of the problems associated with oral diseases [4–7]. The immune system disorders and autoimmune responses, some types of cancer, and possibility of low birth weight (LBW) are other problems associated with oral diseases [4, 5, 8, 9]. In addition to comorbidities, direct and indirect treatment costs related to oral diseases are significant. These costs are usually paid by insurance, government budget, and/or out-of-pocket payments. Nevertheless, 41 countries with different development levels have reported dental costs associated with exposure of families to catastrophic health expenditure [10].

Oral public health services are a part of primary healthcare [1, 2]. Inappropriate prepayment system for risk accumulation and low access to dental health services, particularly in low-income countries, are the main underlying factors for catastrophic expenditures related to dental health services [10–15]. In recent years, some countries (regardless of the type of healthcare system) have been reforming their dental health programs to provide their citizens with a functioning dental insurance package [12, 16–20]. These include compulsory coverage of a larger number of dental health services and provision of free services to some target groups including children, adolescents, pregnant women, and the elderly. Such reforms have led to improved access to services, better quality dental health services, and reduced financial barriers to utilization. Further, an increasing demand to use dental health services has resulted in improving dental indicators and decreasing lost work time related to oral diseases in those settings [19, 21–24].

### The status of dental health services in Iran

The number of people in need of dental health services is high in Iran. One study reviewed the calculated scores for decayed, missing, and filled teeth (DMFT) indices and reported the overall DMFT index was 3.65 with a range of 2.30 to 8.60 across peoples in diverse age groups and health status levels. This finding is unfavorable according to WHO goal of achieving DMFT equal to 1 or lower for the year 2015 [25–28].

Various health system reforms, i.e. “the primary healthcare (PHC) network system”, “public insurance law”, and “health transformation plan (HTP)” have provided some oral health services to the target groups in the course of last four decades [29]. Nevertheless, on average, basic public insurance packages pay only for 3% of costs for basic services such as scaling, simple radiography, surface filling, and tooth extraction, while, complementary insurance bears only 7% dental expenses, and the surplus has been paid out of pocket (OOP) [15, 30].

Evidence shows that functioning insurance coverage can significantly increase the use of dental health services in different age and income groups by reducing perceived prices and costs paid ([15, 20, 31–33], statistic.centinsur.ir). To the best of our knowledge, no evidence exists on households’ preferences for dental insurance in Iran. Citizens in various socio-economic levels have diverse preferences for dental insurance, knowing which can help improve the service delivery processes in line with their needs and preferences. This study seeks to identify the households’ preferences for dental insurance in the capital city of Tehran-Iran.

## Methods

### Setting and sampling

This is a qualitative study. Our participants were the head of household or spouse who had referred to the selected public and private dental centers in Tehran, Iran, and accepted to participate in the interview. We excluded the non-Iranian people from this study.

The multi-stage clustering sampling method was used to select the dental centers. First, we used the findings of the 2nd round of the Urban Health Equity Assessment and Response Tool (HEART) project to rank Tehran districts based on their socio-economic development level [34]. We constructed five socio-economic indicators for ranking. These indicators were obtained by combination of variables: literacy rate (percentage of literate people aged 15 to 49), percentage of households above the poverty line, employment rate (percentage of people aged 15 and over who were employed), average annual household investment expense ratio, average annual household savings ratio, and percentage of households that owned a freezer.

**Fig. 1:**
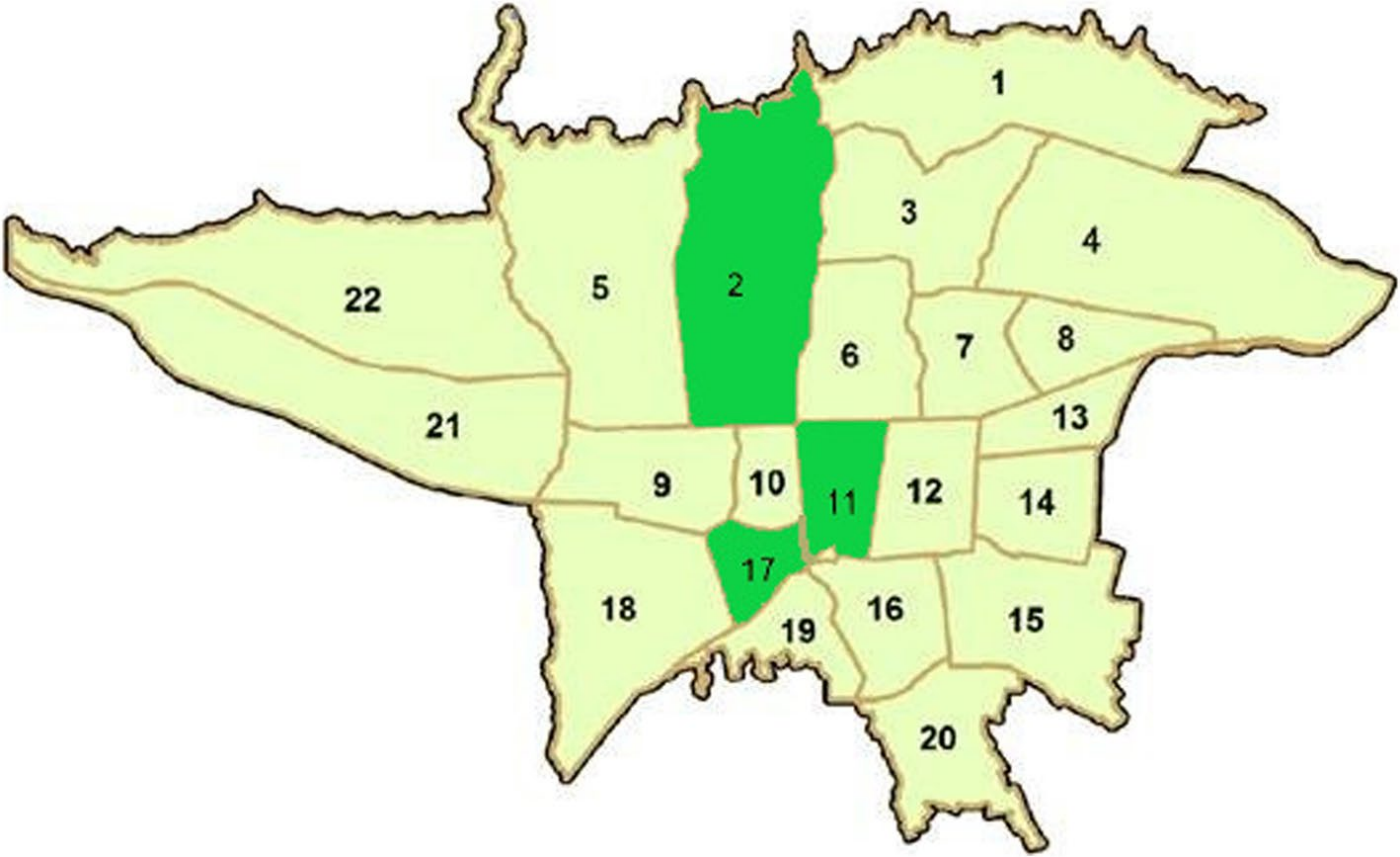
Three districts purposefully selected based on the final ranking of Tehran districts

Each indicator was developed from a combination of variable mentioned above (Additional file 1, S Table 1). We gave identical weight to all the five indicators and calculated the geometric mean for the variables, and then ranked the districts of Tehran city based on each indicator from privileged to non-privileged. Additional file 1 shows this in more detail (Additional file 1, S Table 1). To create a single ranking, we followed several steps. In the first step, district 1 ranked 9 and district 17 ranked 22 since each of these two districts ranked the same based all five indicators. In the next step, the districts that were in the same rank in at least four indicators were ranked and so on. The rank of each district was determined in the ranking table of districts which is shown in the additional file (Additional file 1, S Table 3). Finally, three districts including district 2 (1st rank in the S Table 3), district 11 (at center of the S Table 3), and district 17 (last rank in the S Table 3) were purposefully selected as the most privileged, the average, and the most non-privileged districts of Tehran, respectively (Fig. 1). To select the dental care centers within each district, in line with convenience sampling, we selected an urban comprehensive health service center as a government/public center and a private dental clinic.

### Data collection

In each center, we purposefully selected the interviewees to achieve maximum demographic and economic variation. In total, we conducted 84 semi-structured interviews from October 2018 until January 2019 and continued until we reached saturation. On average, each interview lasted about 25 minutes. Due to lack of access to private clinics in District 17, private clinics in district 19 was purposefully replaced.

The interview guide was initially developed through the review of selected studies on customer preferences for health insurances, and then was reflexively modified after the content analysis of pilot interviews. More information is provided in an additional file (Additional file 2).

Interviews were conducted in person at dental centers by one member of the research team (JV). All interviews were digitally recorded upon the interviewees’ permission, while the interviewer took notes from the key points. All interviews were transcribed verbatim and saved in a Microsoft Word file.

### Data analysis

We used the thematic content analysis method to detect the key attributes associated with households’ preferences for dental insurance and developed a list of main themes and sub-themes. A list of the existing ideas and semantic units was prepared and the basic themes were extracted considering the research objectives, topics raised by the participants, and their opinions and experiences. Comparative analysis was performed by fellow researchers. The key themes and sub- themes were then formulated and named based on codes with similar meanings and MAXQDA Plus version 12 (Release 12.3.0, VERBI GmbH Berlin) was used to assist data management.

## Results

We interviewed 84 participants (as spouses were present in some interviews, 94 people were interviewed in total), of whom 70% were women, 40% had academic education, half were housewives, and 90% were married, 75% received dental health services in their area of residence, 13% had no health insurance, and over 30% had complementary insurance (Table 1).

**Table 1:** Characteristics of study participants

Characteristics		Frequency
Gender	Female	53
	Male	21
	Both (simultaneous interview with household head and spouse)	10
Marital status	Married	75
	Single	6
	Divorced	2
	Widowed	1
Education	Less than high school degree	17
	High school degree (Diploma)	38
	Bachelor’s degree and higher	39
Occupation	Housewife	51
	Private sector employee	10
	Self-employed	16
	Governmental employee	11
	Others	6
Basic health insurance status	No insurance	6
	social security insurance (Obligatory)	47
	Optional social security insurance (Voluntary)	22
	Iranian Health Insurance	4
	Institutional health insurance funds	9
	Others	6
Complementary health insurance status	No	57
	Yes	36
	Not asked	1
Distance of residence place from location of receiving dental health services	Receiving services at neighborhood area	45
	Receiving services at centers not very far from home	24
	Receiving services near the workplace	3
	Others	22
Total		94

The average household size was 3.44. The age group of the interviewees was 22–67 years old and the mean age was 38.22 with the standard deviation of 9.05 (Table 2). The number of referrals for receiving dental treatment services in the last year was 1.7 and the number of referrals for receiving dental prevention services was 0.15.

**Table 2:** Themes, sub-themes and codes related to dental insurance preferences

Theme	Sub-theme	Codes
Pecuniary attributes	Insurance premium	Fixed
		Free of charge
		Varied to type of services
		Varied to salary
	Coinsurance	Between 40 and 70%
		Up to 75%
		Selective
	Insurance coverage granted	Insurance flooring
		Insurance ceiling
		Other coverage granted
	Discounting option	To vulnerable groups
		To low risk individuals
		To low-income insured
	Reimbursement of expenses	Compensating providers
		Compensating patients
Non-pecuniary attributes	Notification status	Enrollment
		Contract renewal
		Centers in contract
	Ethical issues	The insurer
		The insured
		Providers
	Benefits package	Right to choose dentist
		Right to choose dental services
		Inclusion of necessary dental services
	Contract providers	Public provider
		Private provider
		Public/private providers
		Centers in neighborhood
		As enough centers
	Quality of dental centers	Dentist’s work quality
		Less crowded centers
		Shorter waiting time
	Administrative process	Affair process
		Reimbursement process
	Dental insurance scheme	A united package
		An Independent package

According to the thematic analysis, two main themes for choosing a dental insurance were extracted and titled as 1) pecuniary attributes and 2) non-pecuniary attributes. From the identified main themes, 12 sub-themes and 34 sub-sub themes (codes) were extracted (Table 2).

### Pecuniary attributes

Given the growing trend of medical expenses, many interview highlighted the pecuniary attributes among the preferences. The sub-themes of this main theme are described in the following.

#### Sub-theme 1. Insurance premium

According to their experience, income level, and type of services, most participants, especially those interviewed in health centers, preferred a fixed premium. In contrast, the highest premium belonged to private centers.

“I am ready to pay the premium of around 2 million IRR per month for four people... I could not pay something around 10 million IRR.” (District 19 - doctor’s office - interview 7 - married)

Nevertheless, some interviewees preferred dental insurance to be free of charge. With regard to, they believed that dental services should be provided basic insurance. And they demanded the provision of essential dental services through basic insurance without extra premium.

“I think we're paying enough. We pay a monthly premium, but we may not receive any services in return for 2-3 years; so, that is enough.” (District 11, health care center, interview 4, married)

On the other hand, some people were willing to pay more and have better dental insurance coverage with better services:

“We are now paying 4 million IRR per month for four people as the insurance premium. I would be willing to pay up to 5 million IRR per month, but have all dental services covered.” (District 2 - health services center - interview 7 - married)

Many interviewees from deprived background believed that fixed-rate payment was not appropriate for low-income jobs. Whereas, others were concerned that salary-based dental insurance might trouble freelance, informal, and self-employment workers, whose jobs are often without a steady income:

“My husband is self-employed. Complementary health insurance would be very expensive for him. They have to be insured as a group... which is still too expensive.” (District 2 - health services center - interview 9 - married)

#### Sub-theme 2. Coinsurance

The other sub-theme in the pecuniary attributes was coinsurance (the insurer’s contribution to the cost). Noting the significant costs of dental health services and deteriorating economic conditions and high inflation, the interviewees requested at least 40–75% cost contribution by the insurers:

“I think the insurance should offer more, for instance 70%; otherwise, it is pointless” (District 2, clinic, interview 6, married)

Some participants suggested a revised tariff and co-payment mechanism for the most demanding and specialized dental health services.

“It would be better if we might pay above 50% for more basic procedures and the insurance might pay more for specialized procedures.” (District 2, clinic, interview 1, married)

Some interviewees preferred to pay extra premium if they would be able to receive dental services from insurance contract providers:

“Well, I think it was at least 50%. If someone wants to use private centers, they could pay higher percentage.” (District 2, clinic, interview 3, married)

“Insurance companies should have contracts with certain providers; other providers should also accept the insurance at a higher cost. Then, we could choose whether to visit the center parties to the contract or other centers.” (District 11 - dentist's office - interviewer 8 - married)

#### Sub-theme 3. Insurance coverage granted

The insurance coverage granted (coverage limits) was the other sub-them. Some interviewees preferred reimbursement ceiling policy, in a way that the insurance companies bear primary costs, so people will use their insurance benefits to avoid dental restoration. Let alone, some participants complained about lack of trust in the insurance companies as the main reason for this preference:

“It is better that insurance companies pay at first because someone who goes for complementary insurance may have financial problems and cannot afford dental services.” (District 19, clinic, interview 7, married)

Some other interviewees preferred insurance reimbursement for costs above the insurance flooring. Due to their dental diseases and the possibility of heavy costs of treatment, they preferred to pay up to a certain amount of cost, first, and have the rest paid by the insurance.

“I think it’d be great if I could pay up to 10 million IRR and have the compensation for the rest. Most people need the dental care services at least once a year. You'd have to pay 30 million IRR to treat three teeth.” (District 11 - health center - interviewer 3 - married)

“I prefer to pay up to a maximum limit; if the costs exceed the limit, the insurance should pay.” (District 11, clinic, interview 8, married)

A group of interviewees were concerned about the existing insurance reimbursement policy, which leaves big room for OOP due to the high cost of some dental health services and noted its negative consequences. They accused the insurance coverage limits as dysfunctional and inappropriate because dental health costs vary:

“I prefer proportional rate for insurance premium. With parameters such as varied cost of materials and sanctions, it is impossible to have a maximum; so, it is better to be in percentage.” (District 2, clinic, interview 1, married)

#### Sub-theme 4. Discounting option

Most interviewees sought a discounted rate for vulnerable groups, low risk individuals, and the low-income insured, as a government duty that must be paid from the public budget:

“It should be free for children up to the age of seven and for adults above the age of 70. The government should support these people or those with certain diseases.” (District 2, clinic, interview 13, married)

“I think they should give a discount at the beginning of each year, like the car insurance, or consider an incentive for those who pay on time or those who do not use it.” (District 11, clinic, interview 8, married)

However, few participants were happy to pay their insurance premium without discount and accused the discounting policy fraudulent and immoral:

“… unfortunately, everyone wants to use discounts, if possible, whether they are eligible or not.” (District 19, clinic, interview 7, married)

#### Sub-theme 5. Reimbursement of expenses

The last sub-theme in the pecuniary attributes was reimbursement of expenses. This comprised of two codes: compensating providers, and compensating patients by mentioning their experiences in basic/complementary health insurance policies, and high inflation over a period of several months, the interviewees highlighted the importance of determining who is entitled to receive the reimbursement:

“Patient pays a part of the costs; then, the health center could take the rest from the insurance, in the same way as pharmacies. You pay your share and it takes the rest from the insurance.” (District 17, health care center, interview 1, married)

“It should be repaid at the same time. The money loses its value. 4,000 IRR paid this year will worth less than 1000 IRR the next year.” (District 11, clinic, interview 1, married)

### Non- pecuniary attributes

The main non- pecuniary sub-themes identified were explained as notification status, ethical issues, benefits package, contract providers, quality of dental centers, administrative process, and dental insurance scheme. These sub-themes are described in the next paragraphs.

#### Sub-theme 6. Notification status

The notification status was particularly important for a group of interviewees with non-compulsory social health insurance. They expected the insurance company to provide more information about dental centers in contract with the insurance firm:

“It must be integrated. I cannot call everywhere to ask if they accept this particular insurance or do they provide these services?” (District 2, health care center, interview 9, married)

They also felt they needed more guidance and information on registration and contract renewal. Some interviewees preferred to be notified about the expiry date of their contract via a text message. Also, some suggested they opt for automatic renewable in the insurance contract form.

“I wish it’d be automatically renewed. Or a text message could notify us, like the car insurance notifications before the expiry date to see whether we would like to renew the contract. It’d be better this way.” (District 19 - doctor’s office - interview 9 - married)

#### Sub-theme 7. Ethical issues

This sub-theme was the interviewees’ request for greater attention to the management of ethical issues related to the insurers, the insured, and service providers. In particular, participants expressed their concerns about the possible flaws in insurance warranties:

“Insurances must abide by their warranties, so that the insured does not cancel the contract due to the non-fulfillment of warranties.” (District 2, health care center, interview 6, married)

Besides, some requested that the insured records become accessible in appropriate ways to avoid the violation of the rights of other insured persons:

“I shouldn’t be able to [illegally] receive insurance reimbursement, while I receive no dental services for a year.” (District 11, clinic, interview 6, married)

In addition, some interviewees were concerned about the possibility of immoral behaviors of some healthcare providers, e.g., fee splitting and induced demand.

“Unfortunately, there are some immoral behaviors among providers. Something must be done about it. For example, the doctor says: ‘it's 12,000 million IRR, but I’d write 15,000, so that you can get money from the insuring company’.” (District 17 - health center - interviewer 14 - married)

#### Sub-theme 8. Benefits package

The next sub-theme was benefits package and the need to include some preferred services, e.g. the right to choose the dentist, into it, which might lead to a trust in quality of services, peace of mind, and a sense of freedom in those under insurance coverage:

“If there is a system that lets me choose my doctor, well, that would be much better than going to a center determined by the insurance.” (District 2, clinic, interview 1, single)

A number of interviewees cited the inclusion of necessary dental services, mainly the provision of prevention, education, and screening services in benefit package as an appropriate method to reduce future further expenses. In particular, they emphasized the necessity to make periodic visits compulsory:

“I think it must be compulsory. A person who has a contract whose expenses are supposed to be met by insurance must be visited at least once a year or once every 6 months.” (District 11, health care center, interview 1, married)

Some participants believed they were entitled to choose the type of insurance and preferred that this insurance be part of their basic insurance. They wished they had the right to choose to join the insurance and use its benefits.

“I think an insurance program with a right to choose options is better. A type of insurance with a series of basic services, such as tooth extraction, root canal therapy, dental crown, scaling and the rest, should be selected by the insured. If you want to have these, you should pay more.” (District 19, clinic, interview 9, married)

#### Sub-theme 9. Contract providers

This preference included access to required services within public centers, utilizing private centers, the possibility of receiving services in both public and private centers.

“Now, we are going to our local health center for fasting blood sugar and lipid tests. We have a medical record there. They should do dental screening at least once every 6 months or once per year, which could provide counseling and, then, do the referral if needed.” (District 17, health care center, interview 11, married)

“I prefer to pay more to get dental services in the private clinic. It should be what I want.” (District 19, clinic, interview 3, married)

Access to centers in neighborhood and access to as enough centers were the other codes:

“Well, it’s better if it’s close by. It’s difficult to make an appointment if it’s far away. Some hospitals ... also provide dental care services, but we can't often use the services because they are far away.” (District 2 - health services center - interviewee 8 - married)

#### Sub-theme 10. Quality of dental centers

The interviewees expected quality services, less crowded centers, and shorter waiting time to feel they are valued customers:

“I prefer to choose a dentist with high-quality practice and tooth filling. I do not visit a dentist who has poor quality of work.” (District 2 - dentist's office - interviewee 12 - married)

“My time is important for me; the less the crowd, the better the care and attention would be.” (District 2, health care center, interview 1, married)

#### Sub-theme 11. Administrative process

Many interviewees expected easy affair process and easy reimbursement processes, both through a robust electronic health record system and avoiding physical attendance at insurance centers:

“... I'd rather send a photo via my phone ... We have to come a long way and that is very difficult ... It would be much better if we had electronic services.” (District 11, health care center, interview 4, married)

“It would be better if insurance can give us a card to provide services in these centers for financial transaction.” (District 19, clinic, interview 10, married)

#### Sub-theme 12. Dental insurance scheme

While all interviewees emphasized quality of dental health services, they were divided in terms of inclusion of dental health services in their basic health insurance coverage or having a separate dental insurance scheme. This sub-theme was defined as the insurance scheme. The first group thought that a united insurance package can help reduce parallel treatments and extra expenses. In contrast, the second group advocated an independent dental insurance that might lead to better dental health services:

“I prefer a separate insurance scheme, just for dental services…. I think it would be much easier if I had a separate card.” (District 19, clinic, interview 11, married)

“I prefer the health insurance notebook. It avoids task duplication.” (District 19, clinic, interview 9, married)

## Discussion

Oral diseases are common and among the most expensive diseases. Appropriate prepayment system based on the households’ preferences could provide citizens with better access to needed dental health services. We identified two main themes and 12 sub-themes associated to households’ preferences for dental insurance. They included pecuniary attributes (insurance premium, coinsurance, insurance coverage granted, discounting option, reimbursement of expenses), and non-pecuniary attributes (notification status, ethical issues, benefits package, contract providers with health insurance, quality of service centers, administrative process, and dental insurance scheme).

Our findings revealed people’s preference to pay the insurance premium as fixed, salary-based, and based on the service received. Most participants emphasized their reluctance to pay extra premium for dental insurance and stated that their insurance premiums paid for basic and complementary health insurance was enough to cover all the required dental health services. In contrast, among some high-risk households with desire to pay extra premium for dental insurance, they could not afford this due to limited payment capacity. The salary-based payment for insurance premium may persuade some household to buy this insurance. These low-income interviewees assumed that salary percentage-based payment is more cost-effective than fixed premium. A number of studies show that the insurance premium and cost-benefit ratio of insurance premium are important factors in household’s decision-making for enrollment in the insurance plan. For the vulnerable groups, a high insurance premium led to non-purchase of insurance [35]. One study in Malawi showed the insurance premium was one of the three main factors in people’s preferences for choosing insurance and most participants selected the fixed insurance premium [36]. Another study in Ethiopia concluded that the payment of insurance premium was government’s responsibility; but in case of compulsory participation to use private or public sector services, participants were willing to pay 1 to 3% of their salary [37, 38]. Nevertheless, one study in Netherlands revealed more than two-third of people had no desire to pay insurance premium for health services or were willing to choose a program with minimum premium [39].

Payment mechanism was also one of the preferences expressed in our study. A large number of interviewees were willing that at least a half of the costs to be paid by the insurer. The reimbursement of costs below the coverage ceiling was another preference of the participants. In other words, it seems that they planned in such a way to be safe from excessive expenses using the repayment system and insurance risk accumulation. It is noteworthy that some interviewees said the dental health costs are too high and dental health services are usually among the last priorities in the household share. Some household heads said because of high costs, they postponed dental treatment for themselves, and prioritized dental treatment of their children. Postponing dental treatment not only reduces dental health; but also increases costs due to delays in referral. Some interviewees mentioned that less OOP when receiving the services, and raising coverage ceiling encouraged them to pursue needed dental health services. One study in Addis Ababa showed health insurance did not provided suitable financial protection for the insured people [37]. Another study found the low co-payment when receiving service was one of the priorities of the insured [40]. In other studies, non-payment or payment of 50% of the costs when receiving the service was among the preferences of the participants. Some studies revealed that the increasing level of cost coverage to 90% doubled the probability of choosing health insurance [40–42].

Our study showed that a discounted premium and insurance copayment can increase utilization of required dental health services, especially among the vulnerable households. One study in Netherlands found that participants more favored the scenarios with discounts than those that offered lower premiums [43]. In other studies, interviewees were interested in receiving rewards for preventive behaviors and were willing to be paid back a part of yearly premium if they would not have used the insurance during the year [37, 44].

Our study illustrated the participants’ preference to remove the financial relationship between the insured and dental health providers. It is notable that problems with reimbursing the service providers can reduce their level of cooperation and make them reluctant to provide health services to the insured patients. Other studies have shown that due to low repayment rates which usually do not cover high dental procedure costs, and the difficult administrative procedures of the insurer, especially in the case of social insurance, dentists restricted providing services to insured patients, which increased inequality, especially among vulnerable social groups [45, 46].

In the present study, the importance of notification about insurance and its renewal were reported. Inadequate notification seems to make the insured parties unaware of the service centers, time of renewal of the insurance contract, reimbursements, etc., which leads to dissatisfaction, non-renewal of the contract and limited access to the centers and services. For example, people view the service as cost-ineffective or refuse to renew the insurance contract due to not knowing the service centers (in the vicinity of their residence place) which makes them travel long distances to receive the service. Different ways of renewing an insurance contract and notifying about it can make it easier to renew an insurance contract, having the right to choose, feeling satisfied and exploiting the benefits of group insurance. One study found that having guides and liaison bodies that have no conflict of interest is an effective way to help insured parties learn more about their insurance options. In this way, people who run small businesses and uninsured people become familiar with the insurer agents and their services, and learn how to register for insurance, which leads to more equitable distribution of health services [47].

The ethical issues was one of the preferences mentioned in this study. By recounting their experiences, the participants reported immoral behaviors of health providers, the insurers, and insured patients. These immoral behaviors can cause feelings of loss and distrust of other insured persons and spread irresponsible behaviors to compensate for losses and pose ethical problems for various insurance-related groups. Several studies on health insurance plans in other countries have reported immoral behaviors such as over-prescribing medications, unnecessary and repeated visits to health care providers, and receiving payment for services not provided [48]. In one study, Indian insurers stated that they kept their insurance secret until discharge [48]. Nevertheless, a study in Finland reported that there was no evidence in favor of the ethics of the service provider in public or private insurance [49].

Our findings also showed that the insurance benefits package was one of the main factors affecting the choice of insurance, similar to the experience of the study in Malawi that reveled the preference of comprehensive and medium benefits packages to the basic packages. In the age group over 55 and also among high-cost households, these preferences were among the main themes [36, 40]. In other studies, a comprehensive benefits package was preferred and the full dental benefits were among the expected features to select an insurance [37, 38, 43]. Our study highlighted that compulsory dental insurance package needs to consist of periodic visits, screening, root canal, and filling. We also found that many participants do not receive the dental health service, unless their dental diseases might be really serious. The low referral rate for prevention visits also confirmed this finding. The individuals expected to be required to attend compulsory periodic visits, be informed about their dental health status, and receive the services before they become expensive. They stated that filling teeth and root canal services are the most frequent needed services, which are not usually covered by b health insurance. A number of interviewees especially those with better dental health preferred to be allowed to choose a more complete package that covering cosmetic services including orthodontics, teeth whitening, dental crown, implants and surgery. They also preferred to have the right to add or remove dental package to their insurance coverage on the annual basis. We could not identify similar cases in other studies.

Preference on provider of dental services has been less emphasized in other studies. One study in Iran reported that the individuals preferred the provider of dental services in private sector, particularly among those with higher socioeconomic status [41]. Some our study participants preferred to receive services from the private sector, because of easier appointments, shorter waiting time, and perceived better quality. In contrast, some interviewees preferred public centers because of quality of dental services, less cost, and good access. They requested that public facilities, e.g. hospitals and clinics, become more active and provide more dental health services.

Our study also revealed choice of the dentist and the features of service centers were among the preferences. A German study also concluded that the right to physician choice is one of the important insurance features from the viewpoint of the insured [44]. In other studies, the quality of services has been one of the most important preferences for health insurance in those with and without insurance coverage [43, 50].

Administrative process of dental insurance have not received great attention in other studies but were among the priorities of our research. One study in the Netherlands reported the non-complex administrative relationship as one of the preferences of the participants [43]. Some interviewees in our study expected more attention to new technologies and web-based platforms to facilitate the administrative process. The use of electronic health records to connect the insurance centers, upload related documents and follow reimbursement administrative process, were particularly mentioned. Some also suggested providing the insured with a credit card for payments related to dental health services, aiming to reduce the direct costs of printing, travel, and administrative processes, as well as indirect costs i.e. absence from work, dissatisfaction of the insured, traffic and air pollution. In addition, this approach could enhance payment transparency and reduce immoral problems.

Our study found more emphasis on inclusion of dental health services into the social health insurance package, compared to other studies. One study in the northwest of Burkina Faso noted that adding dental health services to community-based health insurance persuaded the rich and the educated to enroll in health insurance [50]. This method can avoid the repeated tasks such as issuing insurance list, issuing separate insurance card, annual survey for continuous membership and renewal, and reviewing applications, while the increasing number of the insured by a comprehensive benefit package will make this insurance cost-effective.

### Implications for policy

The results of this study provided some policy implications:

To develop a more convenient dental insurance benefit package, and promote the citizens’ demand for this insurance package, either policymakers or insurance companies need to consider the citizen’s preference when setting premium, the minimum and maximum benefits, and reimbursement approaches.

Risk accumulation is an advantage of health insurance. Nevertheless, people might get worried about the overuse of dental insurance by some insured persons. We propose rewarding the low-risk potential insured persons, e.g. discounted premium, to persuade them to buy dental insurance.

Our finding suggested that people pay special attention to non-financial aspects of an insurance program. Therefore, policymakers and insurance company could target these aspects in provision of a dental insurance package.

People’s preferences vary regarding the benefit packages of dental insurance. It is vital to consider such diversities for provision of dental insurance packages. It is suggested that insurance companies submit various dental packages to potential consumers and provide them with an opportunity to choose their desirable dental insurance package.

Our study is the first qualitative study to systematically examine the factors influencing the preferences for dental insurance in a sample of Iranian citizens. These findings help inform policy makers to develop dental insurance schemes in a way could encourage more citizens to demand dental insurance. However, the paper has some limitations. Our sampling was restricted to three district of Tehran due to time and financial constraints. Although we tried our best to access a sample of maximum variation by choosing three clusters among all districts ranked based on socio-economic status, findings from this research may not be generalizable beyond the city of Tehran and even to all households in Tehran city. Further qualitative and quantitative studies are needed to validate the findings of this study.

## Conclusion

Qualitative case analysis proved useful for deeply understanding stated preferences of citizen. This qualitative research, conducted in a sample of households in Tehran, revealed that citizens pay attention to different options included in a dental insurance. And, they consider both pecuniary and non-pecuniary attributes for choosing a dental insurance package. This finding also support the importance of providing dental insurance schemes based on citizen’s preference. Our findings could help, we envisage, policymakers to provide a dental insurance scheme that the Iranian households prefer to buy.

## Supplementary Information

**Additional file 1.**

**Additional file 2.**

## Data Availability

The datasets analyzed during the current study are available from the corresponding author on reasonable request. Raw data are not publicly available to preserve individuals’ privacy.
