# Supplementary material for: “To enroll or not to enroll”: a qualitative study on preferences for dental insurance in Iran
Source: BMC Health Serv Res. 2022 Jul 11;22:901. doi: 10.1186/s12913-022-08285-8 (PMC9277837; doi:10.1186/s12913-022-08285-8)
Supplement: Supplementary file 1 — Additional file 1. [file 12913_2022_8285_MOESM1_ESM.docx]

**S Table 1: Socio-economic indicators for ranking**

| Variables | Indicators | | | | |
| --- | --- | --- | --- | --- | --- |
|  | First indicator | Second indicator | Third indicator | Fourth indicator | Fifth indicator |
| literacy rate (percentage of literate people aged 15 to 49) | √ | √ | √ | √ | √ |
| percentage of households above the poverty line | √ |  |  |  |  |
| Employment rate (percentage of people aged 15 and over who were employed) | √ |  |  |  | √ |
| Average annual household investment expense ratio |  | √ |  |  |  |
| Average annual household savings ratio |  |  | √ |  |  |
| Percentage of households who owned a freezer |  | √ | √ | √ | √ |

**S Table 2: The rank of districts of Tehran city based on each indicator**

| Ranking | | | | | Districts of Tehran city |
| --- | --- | --- | --- | --- | --- |
| Fifth indicator | Fourth indicator | Third indicator | Second indicator | First indicator |  |
| 9 | 9 | 9 | 9 | 9 | 1 |
| 1 | 1 | 1 | 1 | 3 | 2 |
| 3 | 3 | 3 | 2 | 7 | 3 |
| 13 | 13 | 13 | 15 | 12 | 4 |
| 4 | 4 | 4 | 5 | 5 | 5 |
| 2 | 2 | 2 | 3 | 6 | 6 |
| 8 | 8 | 8 | 8 | 2 | 7 |
| 7 | 7 | 7 | 7 | 14 | 8 |
| 15 | 15 | 15 | 14 | 13 | 9 |
| 12 | 12 | 12 | 13 | 19 | 10 |
| 14 | 14 | 12 | 11 | 17 | 11 |
| 18 | 18 | 18 | 19 | 21 | 12 |
| 6 | 5 | 6 | 6 | 16 | 13 |
| 10 | 10 | 10 | 10 | 8 | 14 |
| 21 | 21 | 21 | 21 | 15 | 15 |
| 17 | 17 | 17 | 18 | 20 | 16 |
| 22 | 22 | 22 | 22 | 22 | 17 |
| 19 | 19 | 19 | 17 | 10 | 18 |
| 20 | 20 | 20 | 20 | 4 | 19 |
| 16 | 16 | 16 | 16 | 18 | 20 |
| 11 | 11 | 11 | 12 | 11 | 21 |
| 5 | 6 | 5 | 4 | 1 | 22 |

**S Table 3: Ranking table of districts**

| Ranking | Districts |
| --- | --- |
| 1 | 2 |
| 2 | 6 |
| 3 | 3 |
| 4 | 5 |
| 5 | 22 |
| 6 | 13 |
| 7 | 8 |
| 8 | 7 |
| 9 | 1 |
| 10 | 14 |
| 11 | 21 |
| 12 | 10 |
| 13 | 4 |
| 14 | 11 |
| 15 | 9 |
| 16 | 20 |
| 17 | 16 |
| 18 | 12 |
| 19 | 18 |
| 20 | 19 |
| 21 | 15 |
| 22 | 17 |
