# Supplementary material for: “To enroll or not to enroll”: a qualitative study on preferences for dental insurance in Iran
Source: BMC Health Serv Res. 2022 Jul 11;22:901. doi: 10.1186/s12913-022-08285-8 (PMC9277837; doi:10.1186/s12913-022-08285-8)
Supplement: Supplementary file 2 — Additional file 2. [file 12913_2022_8285_MOESM2_ESM.docx]

**Appendix 2:**

**Interview Guide: A qualitative study on preferences for dental insurance in Iran**

- Demographic Characteristics

1. Age of the household’s head
2. Age of the interviewee
3. Occupation status of household’s head (at the time of interview)
4. Education history of the household’s head
5. Marriage status of the household’s head (at the time of interview)
6. Household size
7. Place of location

– Health insurance status

1. Social health insurance status of household’s members and
2. Complementary health insurance status of household’s members
3. Dental package of social and complementary health insurance of household’s members

– Dental health status

1. Dental health status of household’s members during the year before interview (numbers of decayed, missing, and filled teeth)
2. Number of referrals for receiving dental treatment services during the year before the interview
3. Number of referrals for receiving dental prevention services during the year before the interview

| **Focus area** | **Example of questions and probes** |
| --- | --- |
| Dental centers | - What does favorable treatment center mean to you?  - Describe your reasons behind your preference over private dental centers. |
| Dental package | - Describe your preference over the dental services package  - What kinds of dental services do you need?  - How much does your dental services package provided by the health insurance satisfy your needs? |
| Costs Division | - Describe your preference over copayment and franchise.  - Describe your preference over coverage ceiling / coverage roof |
| Bureaucracy | Describe your preference over *bureaucratic* relationship with insurance companies, and dental service providers |
| Patients’ Perception | - How could a dental insurance scheme be improved  - How good/bad experiences have you had with your current health insurance? |
